# Anti-Inflammatory, Antioxidant and Crystallographic Studies of *N*-Palmitoyl-Ethanol Amine (PEA) Derivatives

**DOI:** 10.3390/molecules22040616

**Published:** 2017-04-11

**Authors:** Carmela Saturnino, Ada Popolo, Anna Ramunno, Simona Adesso, Michela Pecoraro, Maria Rosaria Plutino, Silvia Rizzato, Alberto Albinati, Stefania Marzocco, Marina Sala, Domenico Iacopetta, Maria Stefania Sinicropi

**Affiliations:** 1 Department of Science, University of Basilicata, Viale dell′Ateneo Lucano 10, Potenza 85100, Italy; carmela.saturnino@unibas.it; 2 Department of Pharmacy, University of Salerno, Via Giovanni Paolo II n 132, Fisciano (SA) 84084, Italy; apopolo@unisa.it (A.P.); aramunno@unisa.it (A.R.); sadesso@unisa.it (S.A.); mipecoraro@unisa.it (M.P.); msala@unisa.it (M.S.); 3 Institute for the Study of Nanostructured Materials, ISMN-CNR, Palermo, c/o Department of ChiBioFarAm, University of Messina, Vill. S. Agata, Viale F. Stagno d’Alcontres 31, Messina 98166, Italy; 4 Department of Chemistry, University of Milano, Via Golgi 19, Milano 20133, Italy; silvia.rizzato@unimi.it (S.R.); alberto.albinati@unimi.it (A.A.); 5 Department of Pharmacy, Health and Nutritional Sciences, University of Calabria, Via Pietro Bucci, Arcavacata di Rende (CS) 87036, Italy; domenico.iacopetta@unical.it (D.I.); s.sinicropi@unical.it (M.S.S.)

**Keywords:** *N*-acyl-ethanolamines, *N*-palmitoyl-ethanolamine derivatives, biological evaluation of PEA derivatives, anti-inflammatory activity, antioxidant properties, crystallographic studies

## Abstract

*N*-Palmitoyl-ethanolamine (PEA) is an anti-inflammatory component of egg yolk that is usually employed for the prevention of respiratory apparatus virus infection and then frequently used for its efficient anti-inflammatory and analgesic effects in experimental models of visceral, neuropathic, and inflammatory diseases. Nevertheless, data of its use in animal or human therapy are still scarce and further studies are needed. Herein, we report the biological evaluation of a small library of *N*-palmitoyl-ethanolamine analogues or derivatives, characterized by a protected acid function (either as palmitoyl amides or hexadecyl esters), useful to decrease their hydrolysis rate in vitro and prolong their biological activity. Two of these compounds—namely phenyl-carbamic acid hexadecyl ester (**4**) and 2-methyl-pentadecanoic acid (4-nitro-phenyl)-amide (**5**)—have shown good anti-inflammatory and antioxidant properties, without affecting the viability of J774A.1 macrophages. Finally, crystals suitable for X-ray analysis of compound **4** have been obtained, and its solved crystal structure is here reported. Our outcomes may be helpful for a rational drug design based on new PEA analogues/derivatives with improved biological properties.

## 1. Introduction

**Figure 1:**
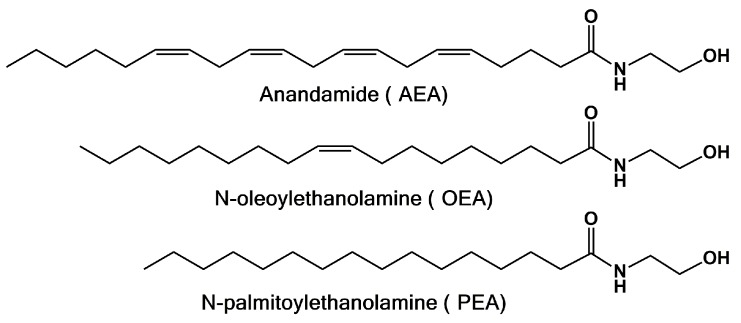
Chemical structures of some of the most studied bioactive *N*-acyl-ethanolamines (NAEs).

*N*-acyl-ethanolamines (NAEs) are well known to be endogenous bioactive amides showing biosynthetic and metabolic pathways [1]. Among this class of compounds, we must mention: (i) *N*-arachidonoyl-ethanolamine (anandamide, AEA, Figure 1), the first endocannabinoid that was discovered; (ii) the anorectic mediator *N*-oleoyl-ethanolamine (OEA); (iii) *N*-palmitoyl-ethanolamine (PEA), known since the late 1950s as an anti-inflammatory component of egg yolk and employed with the brand name of impulsin in the prevention of viral infection of the respiratory apparatus [2,3].

Within the fatty acid ethanolamides, palmitoyl ethanolamide (PEA) has attracted great attention, since it has been found abundantly in the central nervous system (CNS). Its physiological and pharmacological properties have been analyzed, and its therapeutic efficacy in several central nervous system disorders related to pain and inflammation have been thoroughly examined [4].

Nowadays, *N*-palmitoyl-ethanolamine is recognized worldwide for its efficient anti-inflammatory and analgesic effects in experimental models of visceral, neuropathic, and inflammatory diseases, acting through several possible mechanisms. However, so far scarce experimental data have been reported about PEA’s use in animals, or particularly, humans [5].

Under inflammatory and neurodegenerative conditions, the fatty acid amide PEA acts as a “on-demand” protective endogenous mediator to counteract inflammation, neuronal damage, and pain itself.

Some works have demonstrated that, as for the analogous endocannabinoids AEA and 2-arachidonoylglycerol, PEA tissue concentrations are modified during pathological conditions [6].

PEA also appears to be involved in several endogenous mechanisms of protection or in-body response to different types of damage, where a decrease in PEA levels produces an inflammatory reaction.

Furthermore, a decrease in endogenous levels of PEA has been found in the spinal cord in an animal model of neuropathic pain, suggesting that this reduction in response to a damage may affect the inflammatory response [7,8].

Some studies have demonstrated that neuroprotective or anti-inflammatory PEA effects may be due to new G-protein coupled receptors in microglial cells [9] or to PPAR-a [10], respectively, whereas they remain unaffected by cannabinoid receptor antagonists. PPAR-a is a nuclear receptor that plays a key role in lipid nutrient employment and inflammation [10,11]. After an initial activation through ligand binding (which produces conformational changes in receptor conformation), PPAR-a forms a heterodimer formation with the 9-*cis*-retinoic acid receptor, enrolls coactivators and the resulting multiprotein complex, and regulates the transcription of responsive genes. PEA anti-inflammatory effects result from its ability to activate PPAR-a-dependent gene transcription with a time delay of several hours [11,12,13].

Other mechanisms have been proposed to explain PEA’s anti-inflammatory and analgesic effects, such as: (i) Mast-cell degranulation down-regulation via an “autacoid local inflammation antagonism” (ALIA) effect [14]; (ii) the so-called “entourage effect”, which postulates [15,16] that PEA acts by enhancing the anti-inflammatory and anti-nociceptive effects exerted by AEA, often produced together with PEA, and activates cannabinoid (CB) 1 and 2 receptors or the transient receptor potential vanilloid receptor type 1, TRPV1 channels [17,18]. CB1 and CB2 [19,20] are two G-protein coupled receptors sharing 50% sequence identity, and are strongly expressed in the nervous and immune system, respectively [21,22]. The search for CB receptors’ endogenous ligands—so-called “endocannabinoids”—has given rise to the identification of two major classes of lipids capable of activating these receptors; namely, the *N*-acyl ethanolamines (NAEs) and the monoacylglycerols (MAGs) [23,24].

In the search for novel anti-inflammatory drugs related to natural sources (e.g., a series of flavanones isolated from the *Amygdalus lycioides* Spach [25,26]), we thought it worthwhile to prepare promising PEA derivatives/analogues with potential anti-inflammatory and antioxidant activity.

**Figure 2:**
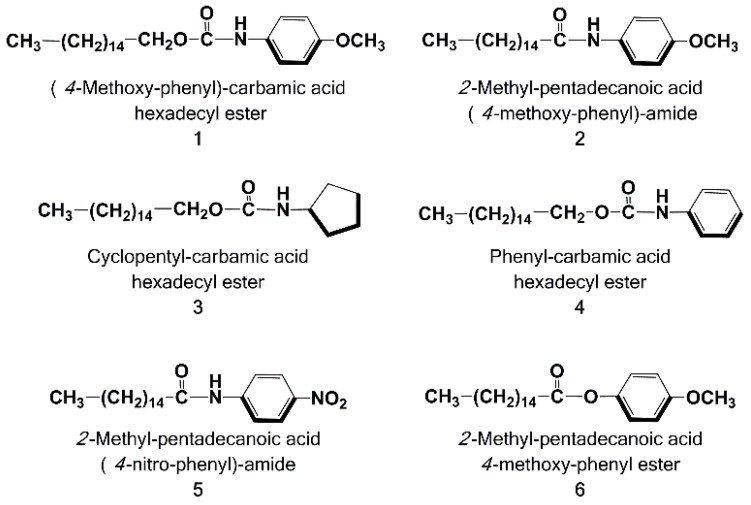
Chemical structures of the employed PEA derivatives/analogues, **1**–**6**.

The aim of this work is the biological evaluation of a small library of PEA analogues or derivatives, **1**–**6**, characterized by a protected acid function, prepared by some of us [27,28], which could bring a decreased hydrolysis rate in a living system (Figure 2). Compound **4**, together with 2-methyl-pentadecanoic acid (4-nitro-phenyl)-amide (**5**), has demonstrated an interesting antioxidant and anti-inflammatory activity. Finally, crystals of phenyl-carbamic acid hexadecyl ester (**4**)—suitable for X-ray analysis—have been obtained, and the solved crystal structure is reported.

## 2. Results and Discussion

### 2.1. Crystal Structure Determination of ***4***

**Figure 3:**
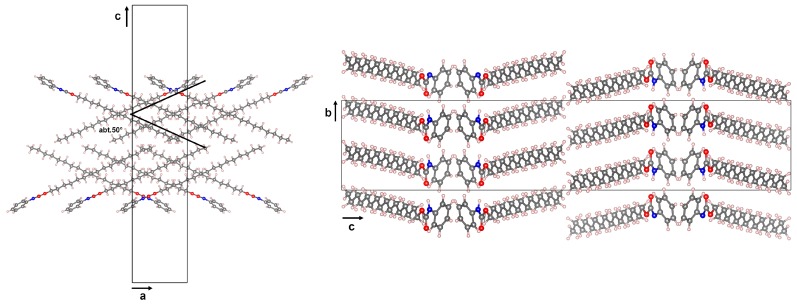
Packing views of the crystal structure of compound **4** down a (**left**) and down b (**right**) axes [29].

Good crystals of **4**, suitable for X-ray diffractometry analysis, have been obtained. An ORTEP view of **4** is given in Figure 3. The PEA derivative **4** crystallizes in the centrosymmetric orthorhombic space group Pbca, with one molecule in the asymmetric unit (Z = 8).

The carbon atoms of the alkyl chain are arranged in a classical zig-zag fashion, with all C–C units staggered and in anti-conformation. The mean plan fitted through the atoms chain have an *rms* deviation of 0.0419 Å and form an angle of 14.5° with the plane fitted through the amide group, which is in turn tilted 21.8° from the phenyl ring.

There is only one strong intermolecular interaction in the crystal structure, which is a hydrogen bond between the amide hydrogen atom of one molecule and the carboxyl oxygen of an adjacent molecule (N-HO 2.849(3) Å) that generates infinite supramolecular chains running along the b axis. The molecules are arranged in parallel, but in two alternating distinct orientations (Figure 3) forming an angle of about 50° degrees.

Very weak van der Waals interactions are also present in the packing, involving the hydrogen atoms of the phenyl groups, while no π–π stacking is observed.

### 2.2. Compounds ***4*** and ***5*** Exert Macrophage Viability

Our data indicated that macrophages viability was not affected by **4** and **5** treatment (data not shown).

### 2.3. Compounds ***4*** and ***5*** Exert Anti-Inflammatory and Antioxidant Activity

To assess the effect of **4** and **5** in influencing NO production, we measured nitrite release—a stable end-product of NO—in cellular medium of lipopolysaccharide (LPS)-stimulated murine macrophage cell line J774A.1 in the presence of **4** and **5** (200–25 µM).

**Figure 4:**
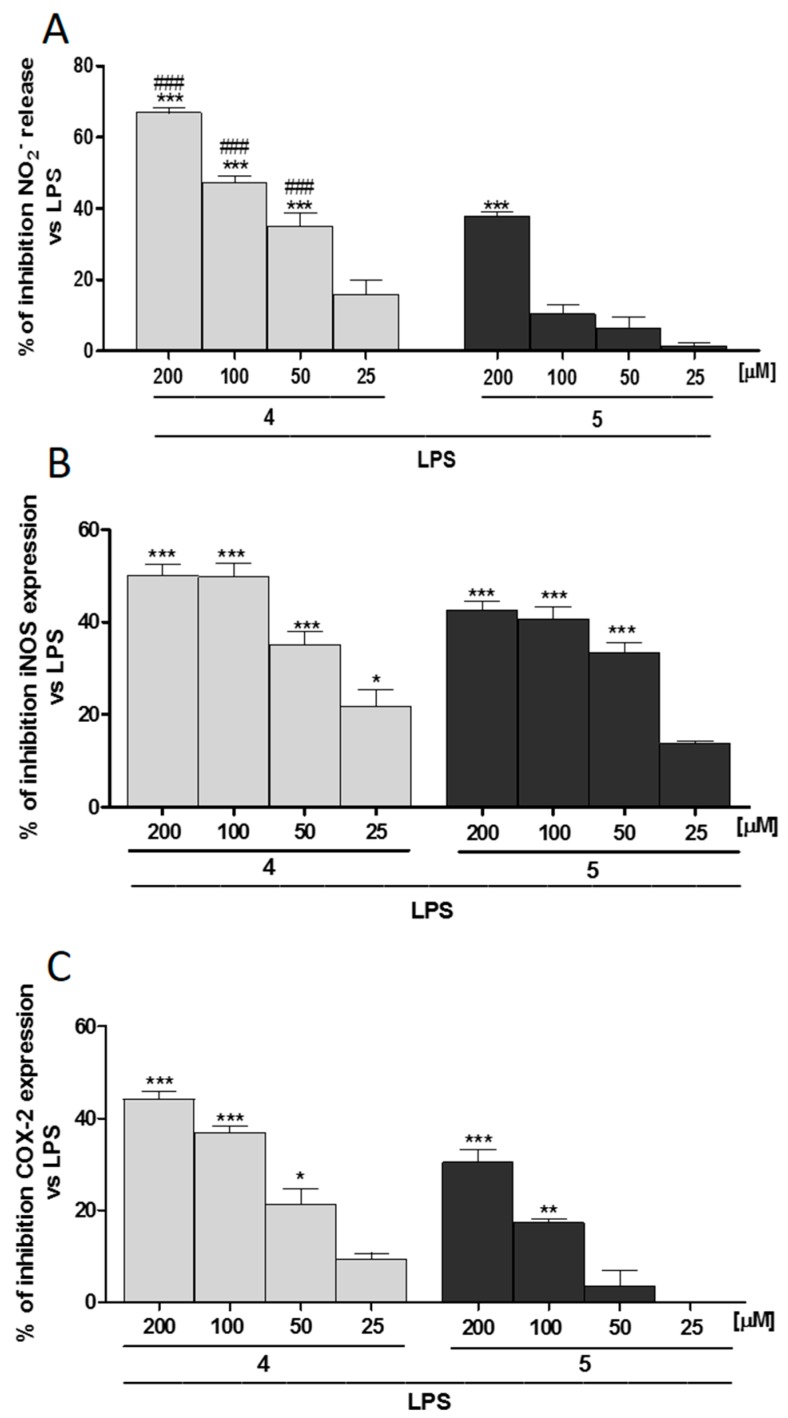
(**A**) Effect of **4** and **5** (200–25 µM) on NO release, evaluated as NO_2_^−^ (µM), by macrophages J774A.1 stimulated with lipopolysaccharide (LPS). Effect of **4** and **5** (200–25 µM) on LPS-induced (**B**) iNOS and (**C**) COX-2 expression in macrophages J774A.1. Values, mean ± S.E.M., are expressed as % of inhibition vs. J774A.1 treated with LPS alone in at least three independent experiments with three replicates each. Comparisons were performed using one-way analysis of variance, and multiple comparisons were made by Bonferroni’s test. ***, **, and * denote *p* < 0.001, *p* < 0.01, and *p* < 0.05 vs. LPS, respectively. ^###^ denotes *p* < 0.001 vs. **5**.

When **4** and **5** (200–25 µM) were added to J774A.1 macrophages 1 h before and simultaneously to LPS (1 µg/mL) stimulation, a significant reduction in NO release in J774A.1 was observed (*p* < 0.001 vs. LPS; Figure 4A).

In particular, **4** significantly inhibited NO in a concentration range of 200–50 µM, while **5** significantly inhibited NO release only at 200 µM (*p* < 0.001 vs. LPS; Figure 4A). Between the two different extracts, **4** exerted the highest inhibitory effect on NO release (*p* < 0.001 vs. **5**; Figure 4A).

To further confirm the observed effect of **4** compared to **5** during LPS-induced inflammation, we evaluate iNOS and COX-2 expression in LPS-treated macrophages. In J774A.1 macrophages, **4** significantly inhibited iNOS at all the tested concentrations (*p* < 0.05 vs. LPS; Figure 4B) and COX-2 expression in a range concentration of 200–50 µM (*p* < 0.05 vs. LPS; Figure 4C). In addition, **5** significantly inhibited iNOS in a µM concentration range (*p* < 0.001 vs. LPS; Figure 4B) and COX-2 expression in a µM concentration range (*p* < 0.001 vs. LPS; Figure 4C).

**Figure 5:**
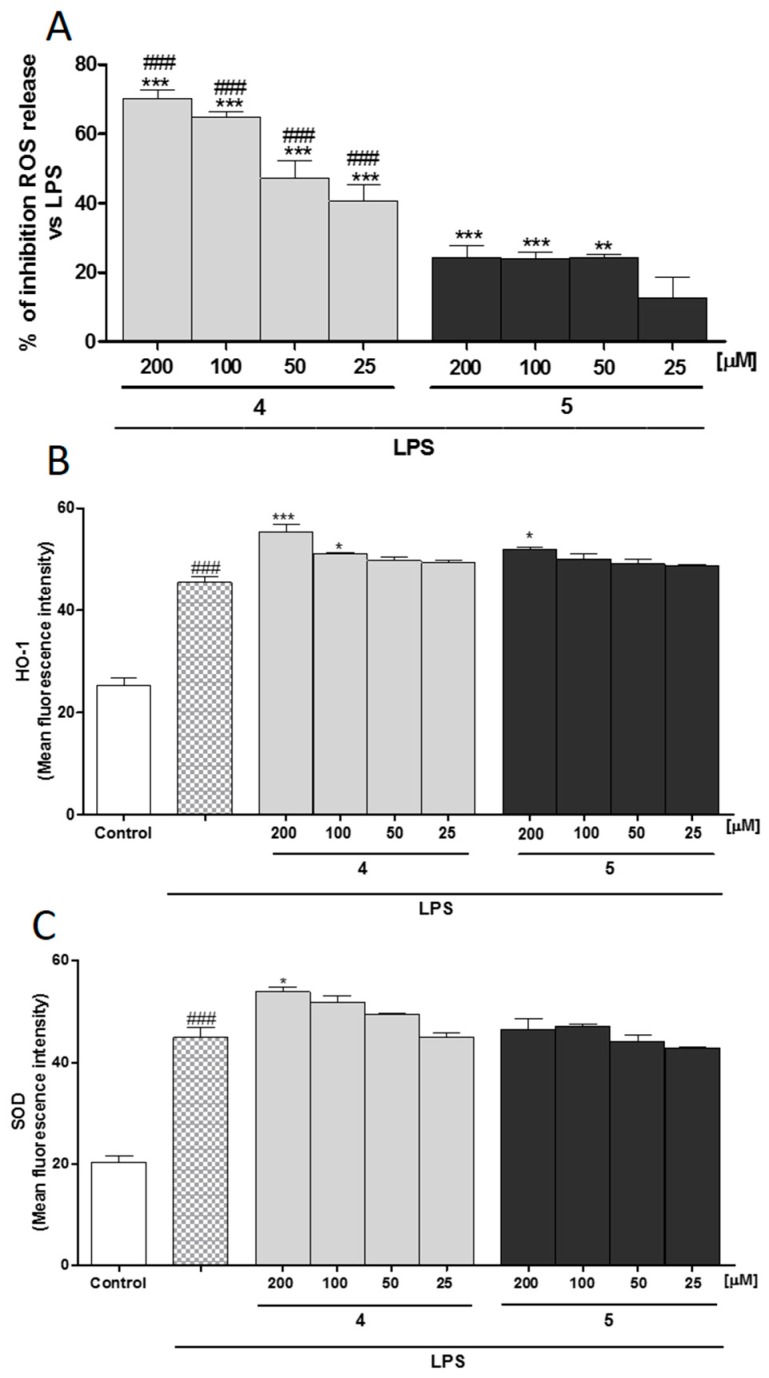
(**A**) Effect of **4** and **5** (200–25 µM) on LPS-induced reactive oxygen species (ROS) in LPS-stimulated J774A.1 macrophages. Effect of **4** and **5** (200–25 µM) on (**B**) LPS-induced HO-1 and on (**C**) LPS-induced Superoxide dismutase SOD expression in macrophages J774A.1. Values, mean ± S.E.M. are expressed as % of inhibition vs. J774A.1 treated with LPS alone and as mean fluorescence intensity of at least three independent experiments with three replicates each. Comparisons were performed using one-way analysis of variance, and multiple comparisons were made by Bonferroni’s test. ***, **, and * denote *p* < 0.001, *p* < 0.01, and *p* < 0.05 vs. LPS, respectively. ^###^ denotes *p* < 0.001 vs. **5**.

When **4** and **5** (200–25 µM) were added to J774A.1 macrophages 1 h before and simultaneously to LPS stimulation, they significantly inhibited ROS production in cell medium (*p* < 0.01 vs. LPS; Figure 5A).

In particular, **4** significantly inhibited ROS release at all the tested concentrations (*p* < 0.001 vs. LPS; Figure 5A), while **5** significantly inhibited ROS release in a concentration range 200–50 µM (*p* < 0.01 vs. LPS; Figure 5A). Between the two different extracts, **4** exerted the highest inhibitory effect on ROS production (*p* < 0.01 vs. **5**; Figure 5A).

In J774A.1 macrophages, both **4** and **5** (200–25 µM) increased HO-1 and SOD-1 expression (Figure 3C and Figure 5B).

In particular, **4** significantly increased HO-1 expression at the concentrations of 100 and 200 µM (*p* < 0.05 and *p* < 0.001 vs. LPS; Figure 5B), while **5** increased HO-1 expression only at the highest concentration (*p* < 0.05 vs. LPS; Figure 5B). Moreover, **4** also increased SOD expression at the highest tested concentration (*p* < 0.05 vs. LPS; Figure 5C).

Compounds **4** and **5** showed an interesting anti-inflammatory potential. Our results indicate that they were able to inhibit NO release by LPS-treated J774A.1 macrophages. NO is a pro-inflammatory mediator released by LPS-treated macrophages, and iNOS is the main isoform involved in NO release during inflammation [30,31]. Interestingly, compounds **4** and **5** also significantly inhibited iNOS expression in macrophages, indicating their inhibitory effect on both the iNOS activity and expression. Moreover, their anti-inflammatory activity was also tested on another important pro-inflammatory enzyme, COX-2. The obtained results indicated that compounds **4** and **5** also significantly inhibit COX-2 expression. Finally, it is noteworthy that ROS are essential mediators both in inflammatory response and in oxidative stress conditions, so we evaluated the effect of the studied compounds on ROS production. Compounds **4** and **5** significantly inhibited ROS production and up-regulated HO-1 expression. HO-1 is the rate-limiting enzyme in heme degradation, and catalyzes the oxidation of heme to generate several biologically-active molecules, such as carbon monoxide (CO), biliverdin, and ferrous ion [32]. HO-1 can increase cellular antioxidant status by generating [33] antioxidants such as bilirubin, which can contribute to an inhibition of iNOS protein expression and a suppression of NO production. HO-1 is normally expressed at low levels in most tissues/organs, except for spleen; however, it is highly inducible in response to a variety of stimuli (e.g., LPS), to protect cells against oxidative and inflammatory injury [34,35].

Taken together, our results indicate that the examined compounds **4** and **5** possess good anti-inflammatory and antioxidant properties, without exerting cytotoxic effects on macrophages.

## 3. Materials and Methods

### 3.1. Chemistry

All chemical reagents were of analytical grade, purchased from commercial suppliers and used as received without further purification, unless otherwise noted. The PEA analogues/derivatives **1**–**6** were prepared by following already-published synthetic procedures [27].

### 3.2. Cell Culture

J774A.1 murine monocyte/macrophage cell line (American Type Culture Collection, Rockville, MD, USA) was grown in adhesion on Petri dishes and maintained with Dulbecco’s modified Eagle’s medium (DMEM) supplemented with 10% fetal calf serum (FCS), 25 mM 4-(2-hydroxyethyl)-1-piperazineethanesulfonic acid (HEPES), 2 mM glutamine, 100 U/mL penicillin and 100 mg/mL streptomycin at 37 °C in a 5% CO_2_ atmosphere.

### 3.3. Viability Assay

Cells (5.0 × 10^4^/well) were plated on 96-well plates and allowed to adhere for 4 h. Thereafter, the medium was replaced with fresh medium, serial dilutions of our compounds (200–25 µM) were added, and cells were incubated for 24, 48, and 72 h [36,37,38]. Mitochondrial respiration—an indicator of cell viability—was assessed by the mitochondrial-dependent reduction of (3-(4,5-dimethylthiazol-2-yl)-2,5-phenyl-2H-tetrazolium bromide) (MTT) to formazan, and cells’ viability was assessed according to the method of Mosmann, as previously reported [39,40]. Briefly, 5 μL of MTT (5 mg/mL) was added and the cells were incubated for 3 h. Thereafter, the cells were lysed and the dark blue crystals solubilized with 100 μL of a solution containing 50% (*v*:*v*) *N*,*N*-dimethylformamide, 20% (*w*:*v*) SDS with an adjusted pH of 4.5. The optical density (OD) of each well was measured with a microplate spectrophotometer (Titertek Multiskan MCC/340, Titertek Instruments Inc., Huntsville, AL, USA) equipped with a 620-nm filter. The viability of the cell line in response to treatment with tested compounds was calculated as: Percent dead cells = 100 − (OD treated/OD control) × 100.

### 3.4. Nitrite Determination

J774A.1 macrophages were seeded into 24-well plates (3.0 × 10^5^ cells/well) and allowed to adhere for 4 h. Thereafter, the medium was replaced with fresh medium and cells were pretreated with **4** and **5** (200–25 µM) for 1 h and then co-exposed to LPS (1 µg/mL) for 24 h. NO generation was measured as nitrite (NO_2_, µM), indicating NO release by cells in the culture medium, as previously reported [41,42]. NO_2_ amounts were measured by Griess reaction. Briefly, 100 µL of cell culture medium were mixed with 100 µL of Griess reagent-equal volumes of 1% (*w*:*v*) sulphanilamide in 5% (*v*:*v*) phosphoric acid and 0.1% (*w*:*v*) naphtylethylenediamine–HCl and incubated at room temperature for 10 min, then the absorbance was measured at 550 nm in a Titertek microplate reader (Dasit, Cornaredo, Milan, Italy). The amount of NO_2_, as µM concentration, in the samples was calculated by a sodium nitrite standard curve.

### 3.5. Measurement of Intracellular ROS

ROS formation was evaluated through the probe 2′,7′-dichlorofluorescein-diacetate (H_2_DCF-DA) as previously reported [43]. H_2_DCF-DA is a non-fluorescent permeant molecule that passively diffuses into cells, where the acetates are cleaved by intracellular esterases to form H_2_DCF, thereby trapping it within the cell. In the presence of intracellular ROS, H_2_DCF is rapidly oxidized to the highly fluorescent 2′,7′-dichlorofluorescein (DCF). Briefly, J774A.1 cells were plated at a density of 3.0 × 10^5^ cells/well into 24-well plates. Cells were allowed to grow for 4 h; thereafter, the medium was replaced with fresh medium, and cells were incubated with **4** and **5** (200–25 µM) for 1 h and then co-exposed to LPS (1 µg/mL) for 24 h. Cells were then collected, washed twice with phosphate buffered saline (PBS), and incubated in PBS containing H_2_DCF-DA (10 µM) at 37 °C. After 45 min, cells’ fluorescence was evaluated using fluorescence-activated cell sorting (FAC Sscan; Becton Dickinson, #342975 BD Biosciences; San Josè, CA, USA) and elaborated with Cell Quest software (BD Biosciences). Data are expressed as mean fluorescence intensity.

### 3.6. Measurement of iNOS, COX-2, HO-1, and SOD Expression by Cytofluorimetry

J774A.1 cells were plated into 96-well plates (5 × 10^4^ cells/well) and treated with **4** and **5** (200–25 µM) for 1 h and then co-exposed to LPS (1 µg/mL) for 24 h. Cells were then collected, washed twice with PBS, and then incubated in fixing solution for 20 min at 4 °C and then incubated in Fix PermSolution for 30 min at 4 °C. Anti-iNOS (e-Bioscience, Dunwoody Park, Atlanta, GA, USA), Anti-COX-2 (e-Bioscience), HO-1 (Santa Cruz Biotechnology, Dallas, TX, USA), and anti-SOD (Santa Cruz Biotechnology) antibodies were then added for a further 30 min. The secondary antibody was added in Fix Solution, and cells’ fluorescence was evaluated using a fluorescence-activated cell sorter (FAC Sscan; Becton Dickinson) and elaborated with Cell Quest software as previously described [44].

### 3.7. Data Analysis

Anti-inflammatory activity data are reported as mean ± standard error mean (S.E.M.) values of independent experiments, performed at least three times, with three or more independent observations in each. Statistical analysis was performed by analysis of variance test, and multiple comparisons were made by Bonferroni’s test. A *p*-value less than 0.05 was considered significant.

### 3.8. Crystal Structure Determination

An air-stable single crystal of the compound was mounted on a glass fiber at a random orientation on a Bruker Smart APEX II CCD diffractometer (Bruker AXS Inc., Madison, WI, USA). The data collection was performed at 293 K by ω-scan method in the interval 2 < θ < 22°. The space group was determined from the systematic absences, while the cell constants were refined at the end of the data collection with the data reduction software SAINT [45]. The collected intensities were corrected for Lorentz and polarization factors and empirically for absorption using the SADABS program [46].

**Figure 6:**
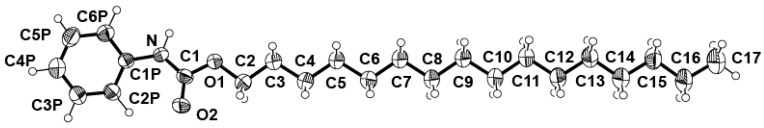
ORTEPIII [50] plot of compound **4** showing the numbering schemes for all non-hydrogen atoms. Ellipsoids drawn at 50% probability.

The structures were solved by direct methods (SIR97) [47] and refined by full-matrix least-squares on F^2^ (SHELXL-97 [48] and WINGX programs [49]) using anisotropic displacement parameters for all atoms except for hydrogens (ORTEP view of **4** is given in Figure 6).

All the hydrogen atoms were located from a Fourier difference map and refined applying a constraint on their isotropic displacements.

The main crystallographic data and structure refinement are collected in Table 1.

## 4. Conclusions

The aim of this study was to perform a screening of PEA derivatives/analogues compounds with potential anti-inflammatory and antioxidant activity. The biological evaluation of compounds **1**–**6** clearly showed that two of them (**4** and **5**) possess good anti-inflammatory and antioxidant properties without affecting the viability of J774A.1 macrophages. Moreover, for the most active compound, **4**, a crystalline structure has been determined. As already shown in the case of receptor compounds employed in the treatment of different CNS pathologies [51], further in vivo studies on these promising compounds are needed to confirm the in vitro observations reported here.

## Figures and Tables

**Table 1:** Crystal data and structure refinement for **4**.

Empirical Formula	C23 H39 N O2
Formula weight	361.55
Temperature	293(2) K
Wavelength	0.71073 Å
Crystal system	Orthorhombic
Space group	*P* b c a
Unit cell dimensions	*a* = 9.6110(10) Å
	*b* = 9.7030(10) Å
	*c* = 48.575(6) Å
Volume	4529.9(9) Å^3^
Z	8
Density (calculated)	1.060 Mg/m^3^
Absorption coefficient	0.066 mm^−1^
F(000)	1600
Theta range for data collection	0.838 to 22.490°
Index ranges	−10 ≤ h ≤ 10, −10 ≤ k ≤ 10, -51 ≤ l ≤ 52
Reflections collected	24670
Independent reflections	2959 [*R*(int) = 0.0472]
Completeness to theta 22°	99.6%
Refinement method	Full-matrix least-squares on *F*^2^
Data/restraints/parameters	2959/0/352
Goodness-of-fit on *F*^2^	1.130
Final R indices [*I* > 2sigma(I)]	*R*1 = 0.0528, *wR*2 = 0.1370
R indices (all data)	*R*1 = 0.0848, *wR*2 = 0.1609
Largest diff. peak and hole	0.188 and −0.195 e·Å^−3^
